# Self-fueling ferroptosis-inducing microreactors based on pH-responsive Lipiodol Pickering emulsions enable transarterial ferro-embolization therapy

**DOI:** 10.1093/nsr/nwad257

**Published:** 2023-09-29

**Authors:** Chunjie Wang, Lei Zhang, Zhijuan Yang, Dongxu Zhao, Zheng Deng, Jialu Xu, Yumin Wu, Yu Hao, Ziliang Dong, Liangzhu Feng, Zhuang Liu

**Affiliations:** Institute of Functional Nano & Soft Materials (FUNSOM), Jiangsu Key Laboratory for Carbon-Based Functional Materials & Devices, Soochow University, Suzhou 215123, China; Center of Interventional Radiology & Vascular Surgery, Department of Radiology, Zhongda Hospital, Medical School, Southeast University, Nanjing 210009, China; Institute of Functional Nano & Soft Materials (FUNSOM), Jiangsu Key Laboratory for Carbon-Based Functional Materials & Devices, Soochow University, Suzhou 215123, China; Department of Interventional Radiology, The First Affiliated Hospital of Soochow University, Suzhou 215006, China; Institute of Functional Nano & Soft Materials (FUNSOM), Jiangsu Key Laboratory for Carbon-Based Functional Materials & Devices, Soochow University, Suzhou 215123, China; Institute of Functional Nano & Soft Materials (FUNSOM), Jiangsu Key Laboratory for Carbon-Based Functional Materials & Devices, Soochow University, Suzhou 215123, China; Institute of Functional Nano & Soft Materials (FUNSOM), Jiangsu Key Laboratory for Carbon-Based Functional Materials & Devices, Soochow University, Suzhou 215123, China; Institute of Functional Nano & Soft Materials (FUNSOM), Jiangsu Key Laboratory for Carbon-Based Functional Materials & Devices, Soochow University, Suzhou 215123, China; Institute of Functional Nano & Soft Materials (FUNSOM), Jiangsu Key Laboratory for Carbon-Based Functional Materials & Devices, Soochow University, Suzhou 215123, China; Institute of Functional Nano & Soft Materials (FUNSOM), Jiangsu Key Laboratory for Carbon-Based Functional Materials & Devices, Soochow University, Suzhou 215123, China; Institute of Functional Nano & Soft Materials (FUNSOM), Jiangsu Key Laboratory for Carbon-Based Functional Materials & Devices, Soochow University, Suzhou 215123, China

**Keywords:** CaCO_3_ nanoparticles, Pickering emulsion, ferroptosis, lipid peroxidation, transarterial ferro-embolization

## Abstract

Lipiodol chemotherapeutic emulsions remain one of the main choices for the treatment of unresectable hepatocellular carcinoma (HCC) via transarterial chemoembolization (TACE). However, the limited stability of Lipiodol chemotherapeutic emulsions would lead to rapid drug diffusion, which would reduce the therapeutic benefit and cause systemic toxicity of administrated chemotherapeutics. Therefore, the development of enhanced Lipiodol-based formulations is of great significance to enable effective and safe TACE treatment. Herein, a stable water-in-oil Lipiodol Pickering emulsion (LPE) stabilized by pH-dissociable calcium carbonate nanoparticles and hemin is prepared and utilized for efficient encapsulation of lipoxygenase (LOX). The obtained LOX-loaded CaCO_3_&hemin-stabilized LPE (LHCa-LPE) showing greatly improved emulsion stability could work as a pH-responsive and self-fueling microreactor to convert polyunsaturated fatty acids (PUFAs), a main component of Lipiodol, to cytotoxic lipid radicals through the cascading catalytic reaction driven by LOX and hemin, thus inducing ferroptosis of cancer cells. As a result, such LHCa-LPE upon transcatheter embolization can effectively suppress the progression of orthotopic N1S1 HCC in rats. This study highlights a concise strategy to prepare pH-responsive and stable LPE-based self-fueling microreactors, which could serve as bifunctional embolic and ferroptosis-inducing agents to enable proof-of-concept transarterial ferro-embolization therapy of HCC.

## Teaser

A stable self-fueling ferroptosis-inducing microreactor constructed by LOX-loaded CaCO_3_&hemin co-stabilized Lipiodol pickering emulsion was rationally developed to suppress the growth of orthotopic N1S1 HCC in rats via transarterial ferro-embolization therapy.

## INTRODUCTION

Hepatocellular carcinoma (HCC) is the main form (∼90%) of liver cancer and is the third most common cause of cancer-related deaths worldwide [1,2]. Due to the difficulty in early diagnosis, most HCC patients are diagnosed in intermediate or later stages and thus lose the opportunity for curative surgical resection and thermal ablation [3–5]. Transarterial embolization (TAE) therapy, which utilizes liquid Lipiodol or polymeric microspheres as embolic agents to induce ischemic necrosis of tumor tissues by blocking their main blood vessels and supplying arteries, is a commonly applied cancer treatment approach for patients with intermediate-advanced stage HCCs [6–13]. In clinical practice, these embolic agents have also been simply combined with chemotherapeutics (e.g. epirubicin, cisplatin) to enable transarterial chemoembolization (TACE) therapy aiming at improved cancer treatments [14–16]. Although Lipiodol emulsion can be applied for the encapsulation of various drugs by principle, its limited stability would lead to the rapid drug diffusion from embolization sites and thus offer limited therapeutic benefits in clinical practice. Alternatively, drug eluting polymeric microspheres are capable of endowing sustained drug release upon intraarterial embolization, but these clinical available ones (e.g. polyvinyl alcohol (PVA) microspheres) can only load weak base chemotherapeutics (e.g. doxorubicin) [17,18]. Aiming at improving TACE therapy, Lipiodol has been formulated into stable Lipiodol emulsions via different approaches to enable sustained drug release in previous studies [19–22].

Ferroptosis is a recently identified nonapoptotic form of programmed cell death which has been intensively explored and shown to kill cancer cells through an iron-dependent lipid peroxidation process [23–25]. Apart from developing small molecular inducers (e.g. erastin and sorafenib), recent progress indicates that tumor-targeted delivery of formulations containing ferric/ferrous ions is an alternative approach to induce ferroptotic cancer cell death by promoting the generation of highly reactive hydroxyl groups (·OH) from endogenous hydrogen peroxides (H_2_O_2_) via the Fenton reaction [26–29]. To avoid the restriction of endogenous H_2_O_2_ concentrations on the ferroptosis-inducing capacity of these ferric/ferrous ion-containing formulations, lipoxygenases (LOXs), an endogenous family of nonheme iron-dependent dioxygenases capable of inducing the oxidation of polyunsaturated fatty acids (PUFAs), have also been explored to initiate cancer cell death [30–32]. Recently, we uncovered that tumor-localized fixation of LOX-based nanoreactors could effectively convert the PUFAs of tumor debris released during radiofrequency ablation to corresponding lipid peroxides and thus suppress the growth of residual cancer cells by causing ferroptosis [33]. Given the existence of abundant PUFAs (e.g. linoleic acid) in Lipiodol [34], we hypothesize that the construction of stable Lipiodol-LOX emulsions is able to suppress the growth of HCCs by concurrently utilizing Lipiodol-supported ferroptosis and embolic effects, defined as transarterial ferro-embolization (TAFE) therapy.

**Scheme 1.**
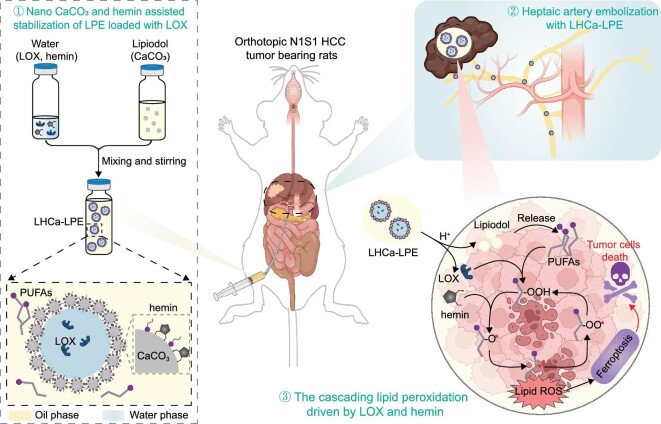
A schematic diagram showing the preparation of a pH-responsive LPE-based microreactor for TAFE treatment of HCC. With CaCO_3_ nanoparticles and hemin as coemulsifiers ①, the yield LPE with excellent stability was capable of initiating a cascading lipid peroxidation chain reaction fueled by Lipiodol itself, thus inducing ferroptosis of cancer cells. Upon transcatheter embolization, it can suppress the progression of orthotopic N1S1 HCCs by serving as a bifunctional embolic ② and ferroptosis-inducing agent ③.

Pickering emulsion is a new type of emulsion utilizing solid nanoparticles instead of organic surfactants as emulsifiers, and such water-in-oil emulsions have showed great capacity in encapsulating various water-soluble agents for different application purposes [35,36]. Herein, calcium carbonate (CaCO_3_) nanoparticles together with amphiphilic hemin were utilized to construct a type of water-in-oil Lipiodol Pickering emulsion (LPE), in which LOX could also be encapsulated during emulsion preparation (Scheme 1). The yielded LOX-loaded CaCO_3_&hemin co-stabilized LPE (LHCa-LPE) showed greatly improved emulsion stability over conventional Lipiodol emulsion, and exhibited pH-responsive release of loaded cargos due to the degradation of nano-CaCO_3_ within the acidic tumor microenvironment. Interestingly, such LHCa-LPE could work as a self-fueling microreactor with Lipiodol itself as the source of PUFAs to generate cytotoxic lipid peroxides via the lipid peroxidation chain reaction driven by LOX and hemin, inducing ferroptosis of tumor cells. Upon intratumoral administration, LHCa-LPE exhibited prolonged retention time and elevated lipid peroxidation, thereby leading to effective suppression of the growth of subcutaneous H22 mouse HCC. More importantly, LHCa-LPE upon being intra-arterially embolized in the tumor region could also effectively inhibit the growth of orthotopic N1S1 HCC in rats, presenting a greatly improved therapeutic outcome compared with TAE therapy alone. Therefore, this study highlights a concise yet robust strategy to prepare stable LPE-based embolic agents with concurrent ferroptotic and embolic treatment effects for the effective treatment of HCC via TAFE therapy, holding great promise for potential clinical translation.

## RESULTS AND DISCUSSION

### Preparation and characterization of LHCa-LPE

**Figure 1.**
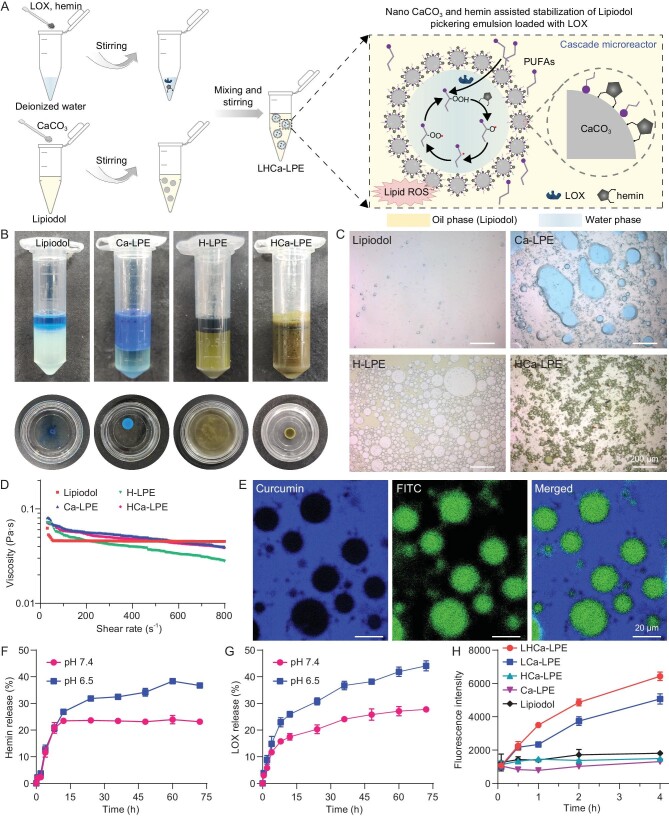
Preparation and characterization of LHCa-LPE. (A) A schematic diagram showing the preparation process of LHCa-LPE and the corresponding emulsifying mechanism. (B) Representative optical images of conventional Lipiodol emulsion, Ca-LPE, H-LPE, and HCa-LPE at 8 h post preparation (upper panel) and corresponding emulsions added to water (lower panel). (C) Optical microscopic images of conventional Lipiodol emulsion, Ca-LPE, H-LPE, and HCa-LPE as indicated. (D) The viscosity of Lipiodol, Ca-LPE, H-LPE, and HCa-LPE recorded under varying vs shear rates as indicated. (E) Confocal microscopic images of LHCa-LPE, LOX and Lipiodol labeled with FITC and curcumin. (F and G) Time-dependent release profiles of hemin (F) and LOX (G) from LHCa-LPE incubated in PBS at pH 7.4 or 6.5 (*n* = 3). (H) Time-dependent lipid peroxidation of various LPEs as indicated (*n* = 3). BODIPY-C11 was pre-dissolved in Lipiodol. Data are presented as the mean ± SD.

To construct pH-responsive and ferroptosis-inducing LPE-based microreactors, we first screened out the optimal parameters for the preparation of stable water-in-oil LPE (Fig. 1A). Consistent with clinical observations, the emulsion of Lipiodol and methylene blue (MB, color indicator) aqueous solution prepared at a volume ratio of 2 : 1 via simple stirring produced a type of water-in-oil LPE with limited stability, indicated by the rapid phase separation and disappearance of water droplets within 30 min both by naked eye and microscopic examinations (Fig. 1B and C, Supporting Fig. S1). Under the same preparation parameters, the addition of CaCO_3_ nanoparticles with a mean diameter of 60.9 nm as observed under scanning electron microscope (SEM) imaging could also produce water-in-oil Ca-LPEs, which showed marked increased stability in a dose-dependent manner (Figs S2 and S3). However, the size of the water droplets exhibited a gradual increase under microscopic examination. While amphiphilic hemin molecules could emulsify Lipiodol to produce oil-in-water H-LPE, the concurrent addition of CaCO_3_ and hemin would lead to the formation of water-in-oil HCa-LPE with further improved stability and a consistent droplet size of 18.5 μm within 8 h (Figs S4 and S5). These results collectively indicate that adsorption of carboxyl group-terminated hemin and free fatty acids in Lipiodol onto CaCO_3_ nanoparticles would increase the surface hydrophobicity of CaCO_3_ nanoparticles, thereby prompting the formation of water-in-oil CaCO_3_-stabilized Pickering emulsions according to previous reports [37,38].

Then, the viscosity of these Lipiodol emulsions was measured by using a rotary rheometer to evaluate their injectability to the hepatic arteries according to previously developed methods [39]. Comparable to that of plain Lipiodol, HCa-LPE exhibited a viscosity of 0.05 Pa·S at a shear rate of 300 s^−1^, corresponding to an injection velocity of 5–10 mL/min in clinical TAE operation (Fig. 1D), indicating satisfactory injectability for TAE operation. Next, we evaluated the capacity of HCa-LPE to encapsulate water-soluble lipoxygenase (LOX). By recording the fluorescence of fluorescein isothiocyanate (FITC) covalently tethered on LOX via confocal microscopy, we found that LOX was located in the water droplets surrounded by the blue fluorescence of hydrophobic curcumin, which was utilized to stain Lipiodol (Fig. 1E). The loading efficiency of LOX was also calculated to be ∼96.7% by recording the fluorescence of FITC. These results indicate that LOX was successfully encapsulated in the water droplets of HCa-LPE, yielding LHCa-LPE microreactors. Considering the presence of pH-dissociable CaCO_3_ nanoparticles within LHCa-LPE, we found that the size of the water droplets within LHCa-LPE incubated at pH 6.5 gradually increased and was obviously larger than that at pH 7.4 under microscopic observation (Fig. S6). As a result, it was shown that LHCa-LPE incubated at pH 6.5 exhibited more rapid release of hemin and LOX compared to that incubated at pH 7.4 (Fig. 1F and G). These results indicate that such LHCa-LPE could enable pH-responsive payload release, thereby promising to potentiate traditional Lipiodol based TACE by enabling sustained drug release at embolization sites.

Lipiodol is an ethylated ester of iodinated fatty acids originally made from poppy seed oil, which has a high fraction of polyunsaturated linoleic acid, α-linolenic acid and other PUFAs [40]. We then carefully assessed the capacity of LHCa-LPE to initiate the conversion of PUFAs to its hydroperoxides and the subsequent cascading lipid peroxidation chain reaction with LOX and hemin as the catalysts (Fig. 1A), by using a commercial lipid peroxidation probe of BODIPY-C11. It was first confirmed that incubation of LOX with Lipiodol solubilized with Pluronic F-127 (PF127) (LOX = 100 μg mL^−1^, Lipiodol = 20 μL mL^−1^) at room temperature induced a time-dependent production of lipid peroxides, as indicated by the gradually increased fluorescence intensity of BODIPY-C11 (Fig. S7). The incubation of hemin (10 μg mL^−1^) with Lipiodol-PF127 solution at room temperature for up to 4 h did not lead to any obvious lipid peroxidation, while the concurrent incubation of Lipiodol-PF127 with LOX and hemin together contributed to the highest level of lipid peroxidation. We then found that LHCa-LPE was the most effective in promoting the lipid peroxidation of Lipiodol via a time-dependent manner as indicated by the gradually increased fluorescence of BODIPY-C11, pre-dissolved in Lipiodol (Fig. 1H). Furthermore, we uncovered that such LHCa-LPE incubated at pH 6.5 was more effective in generating lipid peroxides than that incubated at pH 7.4 (Fig. S8), probably ascribing to the fact that acid incubation would induce the collapse of LHCa-LPE and thus increase the interaction between Lipiodol and the catalytic couple of LOX and hemin. Collectively, these results imply that our LHCa-LPE could work as a type of pH-responsive self-fueling biocatalytic microreactor with Lipiodol itself as the source of PUFAs to induce an effective cascading lipid peroxidation chain reaction driven by LOX and hemin.

### LHCa-LPE induces ferroptotic cancer cell death

**Figure 2.**
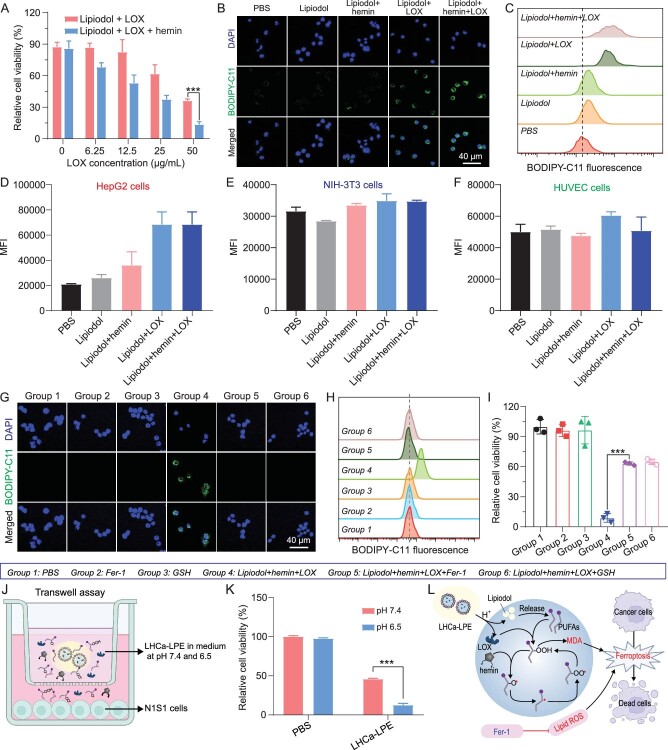
pH-responsive ferroptosis-inducing capacity of LHCa-LPE. (A) Relative cell viabilities of N1S1 cells with various treatments as indicated. (B and C) Confocal images (B) and flow cytometric analysis (C) of intracellular lipid peroxidation of N1S1 cells post various treatments as indicated. (D–F) The mean fluorescence intensity of HepG2 cells (D), NIH-3T3 cells (E), and HUVECs (F) after various treatments as indicated, followed by BODIPY-C11 staining (*n* = 3). (G and H) Representative confocal imaging (G) and flow cytometric analysis (H) of intracellular lipid peroxidation of N1S1 cells post various treatments as indicated. (I) Relative cell viabilities of N1S1 cells with different treatments as indicated. (J) A schematic diagram showing the experimental process for evaluating the pH-responsive cell killing ability of LHCa-LPE by transwell assay. (K) Relative cell viability of N1S1 cells treated with PBS and LHCa-LPE at pH 6.5 and 7.4 for 24 h. (L) Schematic illustration of the mechanism of LOX fueled by Lipiodol in the presence of hemin in inducing cancer cell death. Data are presented as the mean ± SD; *p* values were calculated by using unpaired *t* tests. ***P < 0.001.

We investigated the potency of LOX and hemin with Lipiodol as the source of PUFAs to kill cancer cells by inducing intracellular lipid peroxidation. Considering that LHCa-LPE could not be homogenously distributed inside the cell culture medium, and thus impair the accuracy of the cellular experimental results, Lipiodol was therefore emulsified with PF127 in the form of nanoscale micelles with a mean diameter of 380.7 nm for subsequent cellular experiments (Fig. S9). Via the standard cell viability assay, it was first determined that more than 83.3% of rat N1S1 HCC cells remained alive after incubation with Lipiodol micelles at a concentration lower than 12.5 μL mL^−1^ for 24 h, and hemin or LOX at a concentration lower than 200 μg mL^−1^ also showed negligible disturbance of cell viability (Figs S10–S12). However, under fixed incubation concentrations of Lipiodol (12.5 μL mL^−1^) and hemin (12.5 μg mL^−1^), the addition of LOX led to a concentration-dependent cell killing ability (Fig. 2A). The remaining viability of N1S1 cells incubated with LOX (50 μg mL^−1^) together with Lipiodol and hemin was only approximately 13.6%, which was significantly lower than the 36.2% of cells treated with LOX and Lipiodol.

Next, their capacity to induce intracellular lipid peroxidation was evaluated with BODIPY-C11 as the fluorescent probe via confocal microscopy and flow cytometry. Consistent with the results of the cell viability assays, N1S1 cells incubated with hemin and LOX in the presence of Lipiodol showed effective intracellular lipid peroxidation, which was much higher than that in cells treated with LOX and Lipiodol (Figs 2B, 2C and S13). In contrast, N1S1 cells incubated with hemin and Lipiodol or Lipiodol alone showed negligible intracellular lipid peroxidation. The high potency of concurrent incubation of hemin, LOX and Lipiodol to promote intracellular lipid peroxidation was further confirmed with 2′,7′-dichlorodihydrofluorescein diacetate (DCFH-DA) as the fluorescent probe via both confocal microscopy and flow cytometry (Fig. S14). Additionally, treatment with concurrent incubation with LOX, hemin and Lipiodol was also effective in inducing intracellular lipid peroxidation and cell death in human HepG2 HCC cells (Figs 2D and S15), while negligibly disturbing the intracellular lipid peroxidation and viability of normal cells, including murine NIH-3T3 fibroblast cells (Figs 2E and S16) and human umbilical vein endothelial cells (HUVECs) (Figs 2F and S17). Therefore, these results indicate that LHCa-LPE has the potential to selectively kill cancer cells by inducing intracellular peroxidation, probably because cancer cells are more sensitive to the amplification of oxidative stress than normal cells [41,42].

As the formation of lipid peroxides and malondialdehyde (MDA) is a typical hallmark of ferroptotic cell death, we then carefully studied whether LHCa-LPE was able to induce cancer cell death via the ferroptosis pathway with ferrostatin (Fer-1) and glutathione (GSH) as inhibitors of ferroptosis according to previous studies [43]. Via both confocal microscopy and flow cytometry as mentioned above, it was revealed that the BODIPY-C11–specific fluorescence of N1S1 cells with concurrent incubation of LOX, hemin and Lipiodol was almost fully suppressed upon addition of both Fer-1 (10 μM) and GSH (1 mM) (Figs 2G, H and S18). Additionally, the combined treatment was shown to be capable of promoting the accumulation of malondialdehyde, a downstream product of lipid hydroperoxide, in N1S1 cells, while the addition of Fer-1 and GSH could efficiently inhibit intracellular production of malondialdehyde (Fig. S19). The addition of Fer-1 and GSH could also effectively alleviate the cytotoxic effect induced by the concurrent incubation of LOX, hemin and Lipiodol (Fig. 2I). The above results indicated that LHCa-LPE is capable of inducing ferroptotic cancer cell death, which is attributed to the production of lipid peroxides and malondialdehyde during the reaction process of linoleic acid and α-linolenic acid existing in Lipiodol catalyzed by LOX and hemin (Scheme S1). Furthermore, via the transwell assay (Fig. 2J), it was shown that the cell viabilities of N1S1 cells incubated with LHCa-LPE at pH 6.5 and 7.4 for 24 h were 12.6% and 45.8% (Fig. 2K), respectively, revealing its superior pH-responsive cell killing ability. Taken together, these results reveal that LHCa-LPE is capable of inducing ferroptosis in N1S1 cells by initiating cascading intracellular lipid peroxidation in a pH-responsive manner (Fig. 2L).

### *In vivo* therapeutic efficacy of LHCa-LPE on subcutaneous H22 tumors

**Figure 3.**
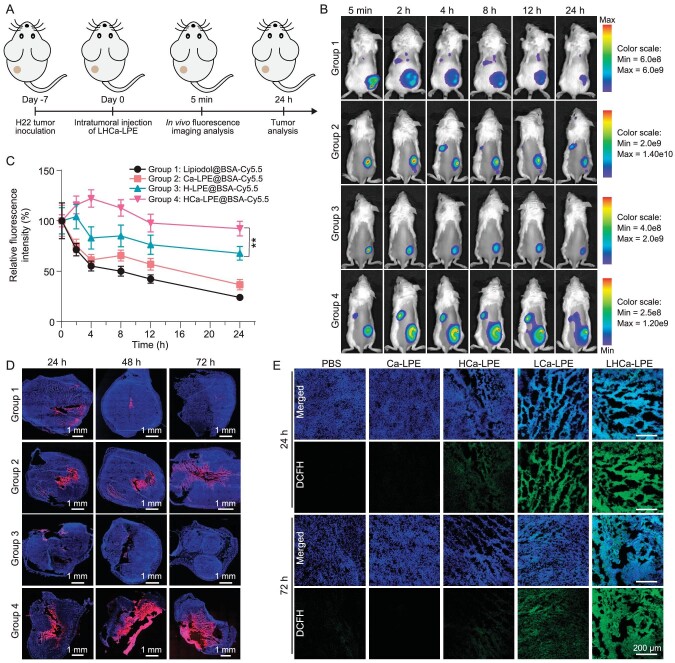
Intratumoral retention and lipid peroxidation potencies of LHCa-LPE. (A) A schematic diagram of experimental schedule. (B) *In vivo* fluorescence imaging of H22 tumor-bearing mice post intratumoral injection of Cy5.5-labeled Lipiodol, Ca-LPE, H-LPE, and HCa-LPE at the indicated time points. (C) Relative fluorescence intensity of H22 tumor area based on the images shown in Fig. 3B. (D) Confocal images of tumor slices collected from H22 tumor-bearing mice with intratumoral injection of Cy5.5-labeled Lipiodol, Ca-LPE, H-LPE, and HCa-LPE at the indicated time points. (E) Confocal images of tumor slices collected from H22 tumor-bearing mice at 24 h and 72 h post various treatments as indicated and stained with DCFH-DA. Data are presented as the mean ± SD; *p* values were calculated by using unpaired *t* tests. **P < 0.01.

In view of the superior stability of HCa-LPE, we next evaluated its capacity to enable the intratumoral retention of physically encapsulated Cy5.5-labeled bovine serum albumin (Cy5.5-BSA) utilized as a representative LOX in subcutaneous H22 tumor-bearing mice (Fig. 3A). By recording the fluorescence of Cy5.5 using an IVIS^®^ Lumina III *in vivo* fluorescence imaging system, H22 tumors in mice with intratumoral injection of Cy5.5-BSA–labeled HCa-LPE showed gradually decreased Cy5.5 fluorescence over a 24 h monitoring process (Fig. 3B). In comparison, intratumoral injection of Cy5.5-BSA–labeled Ca-LPE exhibited similar slow decay of Cy5.5 fluorescence in the tumor region, while intratumoral injection of Cy5.5-BSA–labeled H-LPE and conventional Lipiodol emulsions led to a much quicker decay of Cy5.5 fluorescence. By semiquantitative analysis, it was calculated that ∼71.7% of Cy5.5-BSA encapsulated with HCa-LPE remained in the tumor region at 24 h post intratumoral injection, while 56.6%, 43.4%, and 27.3% of Cy5.5-BSA encapsulated with Ca-LPE, H-LPE, and conventional Lipiodol emulsions, respectively, remained in the tumor region (Fig. 3C). The superior capacity of HCa-LPE to promote the long-term retention of Cy5.5-BSA was further confirmed by recording the Cy5.5 fluorescence in tumor slices via confocal microscopy (Figs 3D and S20). Collectively, these results reveal that HCa-LPE could enable long-term retention of its cargos inside the tumors due to its superior stability to other emulsions.

Next, we investigated the potency of LHCa-LPE to induce lipid peroxidation of tumor cells post intratumoral injection. To this end, five groups of H22 tumor-bearing mice (110 mm^3^) were treated as follows: group I: PBS; group II: Ca-LPE; group III: HCa-LPE; group IV: LCa-LPE; and group V: LHCa-LPE. The doses of hemin, LOX, CaCO_3_, and Lipiodol for corresponding injections were 10 mg kg^−1^, 25 mg kg^−1^, 25 mg kg^−1^, and 1.75 mL kg^−1^, respectively. At 24 h and 72 h post various treatments, these mice were sacrificed, and their tumors were collected and cryosectioned for DCFH-DA staining. As visualized under confocal microscopy, the DCFH fluorescence of tumor slices of mice with LHCa-LPE treatment was shown to be comparable to that of mice with LCa-LPE treatment at 24 h posttreatment but significantly higher than that of tumor slices with LCa-LPE at 72 h posttreatment (Figs 3E and S21). In contrast, tumor slices with the other treatments showed minimal DCFH fluorescence signals at both detection time points. These results collectively demonstrate that our LHCa-LPE could effectively induce intratumoral lipid peroxidation.

**Figure 4.**
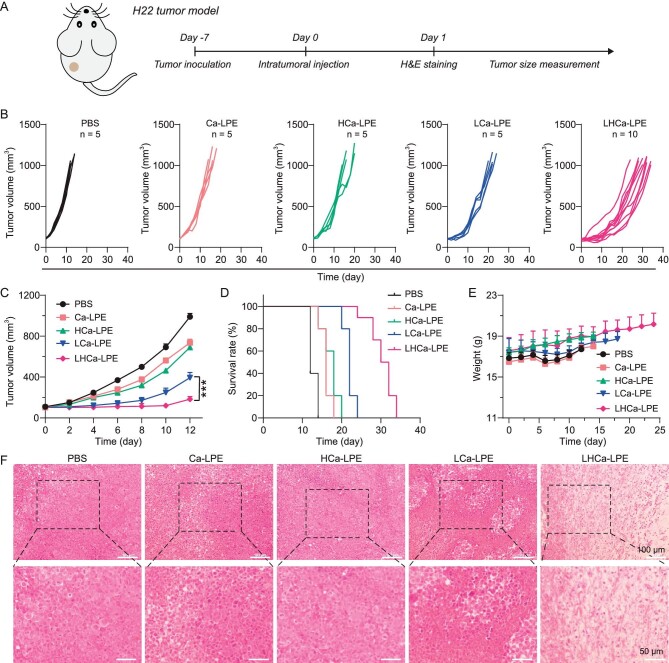
*In vivo* therapeutic potency of LHCa-LPE in H22 tumor model. (A) A schematic diagram of the therapeutic schedule. (B–E) Individual tumor growth curves (B), average tumor sizes (C), mobility-free survival rate (D), and average body weights (E) of H22 tumor-bearing mice after receiving various treatments as indicated (PBS, Ca-LPE, HCa-LPE, and LCa-LPE, *n* = 5; LHCa-LPE, *n* = 10). (F) H&E staining of tumor slices collected from the treated mice at 24 h post various treatments. Data are presented as the mean ± SD; *p* values were calculated by using unpaired *t* tests. ***P < 0.001.

After that, another five groups of mice bearing H22 tumors received the same treatments as mentioned above and were used to evaluate the therapeutic efficacy of LHCa-LPE (Fig. 4A). By recording tumor sizes, it was revealed that the growth of H22 tumors in mice treated with LHCa-LPE was the most effectively suppressed, while these other treatments only showed a moderate inhibitory effect on tumor growth (Fig. 4B and C). The median survival time of mice treated with LHCa-LPE was 31 days, significantly longer than the 22 days, 18 days, 16 days, and 12 days of mice treated with LCa-LPE, HCa-LPE, Ca-LPE, and PBS, respectively (Fig. 4D). It was also shown that no significant body weight change was observed in all treated mice, indicating the excellent biocompatibility of our proposed emulsions at our tested dosage (Fig. 4E). Furthermore, the superior therapeutic efficacy of LHCa-LPE treatment was also carefully evaluated by analyzing the tumor cell damage levels at day 1 posttreatment using hematoxylin and eosin (H&E) staining. We found that these tumor slices collected from the mice treated with LHCa-LPE exhibited the most severe cell damage, while those of Ca-LPE–, HCa-LPE–, and LCa-LPE–treated tumors showed moderate cell damage compared to that of untreated mice (Fig. 4F). Collectively, these results demonstrate that LHCa-LPE can effectively suppress the growth of H22 tumors without inducing obvious side effects.

### *In vivo* TAFE therapeutic efficacy of LHCa-LPE on orthotopic N1S1 tumors

Inspired by the superior stability and tumor suppression capacity of LHCa-LPE, we first explored the effect of LHCa-LPE in inducing lipid peroxidation of orthotopic N1S1 tumors post transarterial embolization with various agents. These orthotopic N1S1 tumor models were inoculated by directly injecting N1S1 cells into the left lobe of the liver, and seven days later, the mice were treated as follows: group I, control; group II, transarterial embolization with Lipiodol; group III, transarterial embolization with LCa-LPE; and group IV, transarterial embolization with LHCa-LPE. The doses of hemin, LOX, CaCO_3_, and Lipiodol were 0.609 mg, 1.5 mg, 1.523 mg, and 150 μL per mouse, respectively. At 3 days post various treatments, N1S1 tumor-bearing rats were sacrificed and their tumors collected and cryosectioned for BODIPY-C11 staining. We found that tumor slices of LHCa-LPE–treated rats showed the highest level of BODIPY-C11 fluorescence compared to that of mice with other treatments (Fig. S22). These results indicate that our LHCa-LPE treatment is able to induce ferroptosis of N1S1 cancer cells by inducing intratumoral lipid peroxidation.

**Figure 5.**
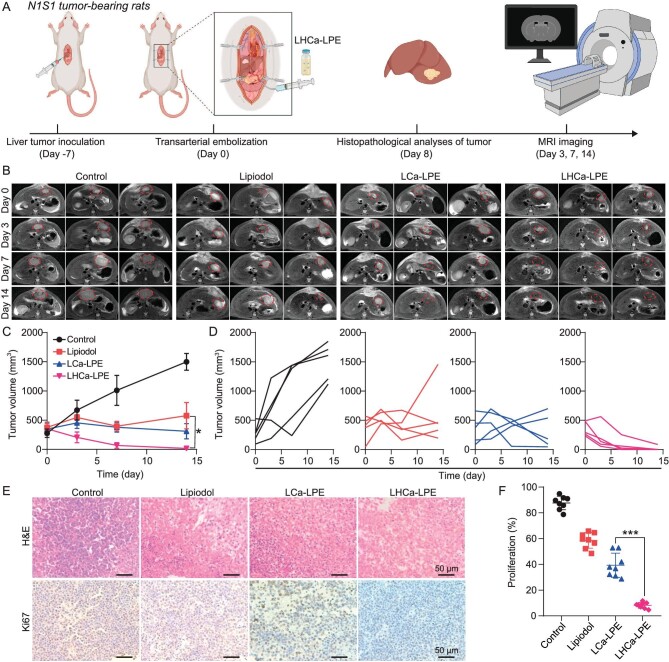
*In vivo* TAFE treatment with LHCa-LPE in an orthotopic N1S1 rat tumor model. (A) A schematic diagram of the TAFE treatment schedule in N1S1 bearing rat. (B) Representative T2 contrast-enhanced MRI scanning of N1S1-bearing rats with different treatments as indicated. (C and D) Average (C) and individual tumor growth curves (D) of different groups of N1S1 tumor-bearing rats after various treatments as indicated. (E) H&E and Ki67 staining of tumor slices collected from these N1S1 tumor-bearing rats with various treatments as indicated. (F) Tumor proliferation inhibition rate of N1S1 liver tumors from different groups based on Ki67-stained slices. Data are presented as the mean ± SD; *p* values were calculated by using unpaired *t* tests. *P < 0.05, ***P < 0.001.

Furthermore, another four groups of rats bearing N1S1 orthotopic HCCs were established and received the same treatments as mentioned above, and were used to investigate the TAFE treatment efficacy of LHCa-LPE (Fig. 5A). At day 0 before various treatments and 3, 7, and 14 days post various treatments, these rats were intraperitoneally injected with commercial gadolinium contrast agent and then subjected to a 3.0-T magnetic resonance (MR) imaging system for recording tumor volumes. Treatment with LHCa-LPE showed the most effective tumor inhibitory effect, with all tumors disappearing at day 14 post corresponding treatment under MR imaging. Treatments with LCa-LPE and Lipiodol alone only partially delayed tumor growth (Fig. 5B–D). Although the body weight of all treated rats showed an ∼10% decrease in the first 3 days postembolization treatment, it gradually increased and returned to normal within the next 11 days compared to the control group (Fig. S23). Then, to further confirm the efficient therapeutic outcomes post TAE therapy, histopathological analyses of H&E and Ki67 staining were conducted to evaluate the tumor damage and cell proliferation statuses of the tumor slices collected from various treated mice (Fig. 5E). TAFE treatment of LHCa-LPE resulted in the most severe tumor damage and the lowest cell proliferation level compared to those of rats in the bare Lipiodol-based TAE group and untreated group (Fig. 5F), further evidencing the significant therapeutic outcomes of LHCa-LPE.

## CONCLUSION

In this study, a tumor acidity-responsive ferroptosis-inducing microreactor was concisely prepared by encapsulating LOX within LPE stabilized in combination with pH-dissociable CaCO_3_ nanoparticles and hemin, thus enabling proof-of-concept TAFE therapy of HCC tumors. It was shown that amphiphilic hemin could assist CaCO_3_ nanoparticles in stabilizing the water-in-oil LPE, as it can significantly increase the surface hydrophobicity of CaCO_3_ nanoparticles through strong coordination interactions between its carboxyl groups and Ca^2+^. Meanwhile, it can work as an efficient Fenton catalyst to promote the propagation of linoleic hydroperoxides initially produced from Lipiodol by LOX, thereby enabling a cascading lipid peroxidation chain reaction to induce ferroptosis of cancer cells. Consequently, the resulting LHCa-LPE not only significantly suppressed the growth of subcutaneous H22 tumors in mice but also exhibited a synergistic therapeutic outcome in orthotopic N1S1 tumors via both embolic and ferroptosis effects. Therefore, this study highlights the concise preparation of a type of pH-responsive LPE-based microreactor capable of initiating a cascading lipid peroxidation chain reaction fueled by Lipiodol itself, thus enabling a proof-of-concept TAFE treatment strategy for potential HCC patients. Given the excellent biocompatibility of all components used for the preparation of such emulsions, TAFE treatment with LHCa-LPE holds great promise for future clinical translation.

## Supplementary Material

nwad257_Supplemental_File
