# Supplementary material for: Self-fueling ferroptosis-inducing microreactors based on pH-responsive Lipiodol Pickering emulsions enable transarterial ferro-embolization therapy
Source: Natl Sci Rev. 2023 Sep 29;11(1):nwad257. doi: 10.1093/nsr/nwad257 (PMC10727844; doi:10.1093/nsr/nwad257)
Supplement: nwad257_Supplemental_File [file nwad257_supplemental_file.pdf]

Supporting information

Self-fueling ferroptosis-inducing microreactors based on pH-responsive Lipiodol pickering emulsions enable transarterial ferro-embolization therapy

Chunjie Wang[1,#], Lei Zhang[2,#], Zhijuan Yang[1], Dongxu Zhao[3], Zheng Deng[1], Jialu Xu[1], Yumin Wu[1], Yu Hao[1], Ziliang Dong[1], Liangzhu Feng[1,*] and Zhuang Liu[1,*]

[1]Institute of Functional Nano & Soft Materials (FUNSOM), Jiangsu Key Laboratory for Carbon-Based Functional Materials & Devices, Soochow University, Suzhou 215123, China;

[2]Center of Interventional Radiology & Vascular Surgery, Department of Radiology, Zhongda Hospital, Medical School, Southeast University, Nanjing 210009, China;

[3]Department of Interventional Radiology, The First Affiliated Hospital of Soochow University, Suzhou, 215006, China

*__Corresponding authors__. E-mails: zliu@suda.edu.cn; lzfeng@suda.edu.cn

Materials and methods

Materials

Nano $CaCO_3$ power was purchased from Nanjing XFNANO Materials Technology Co., Ltd. Hemin, lipoxygenase (LOX), and 2′,7′-dichlorodihydrofluorescein diacetate (DCFH-DA) were obtained from Sigma-Aldrich. Lipiodol was available from Jiangsu Hengrui Pharmaceutical Company. BODIPY-C11 probe was purchased from ThermoFisher Scientific China Co., Ltd. Cell counting kit 8 (CCK8) was obtained from DOJINDO Laboratories. Fetal bovine serum (FBS, BS-1105) was purchased from Inner Mongolia Opcel Biotechnology Co., Ltd. RPMI 1640 medium and DMEM high glucose medium were obtained from Hyclone Laboratories.

Preparation and characterization of Lipiodol, Ca-LPE, H-LPE, and HCa-LPE

Lipiodol emulsions were prepared by mixing deionized water with Lipiodol at a volume ratio of 1 : 2 under the magnetic stirring (5 min). To prepare LHCa-LPE, $CaCO_3$ nanoparticles were first mixed with Lipiodol (0.66 mL) under stirring for 3 min, followed by being mixed with sodium bicarbonate solution (25 mM, 0.33 mL) of LOX and hemin (1 mg, 2 mg, 4 mg) under magnetic stirring. Ca-LPE and H-LPE were prepared via the same protocol with addition of corresponding agents.

To evaluate the stability of Lipiodol, Ca-LPE, H-LPE, and HCa-LPE, their phase separation profiles were monitored by using a digital camera at determined intervals. Their microscopic morphologies were obtained by using a Leica fluorescence optical microscope. Their viscosities were measured by using a rotary rheometer (HAAKE MARS 40).

pH responsive release of hemin and LOX from LHCa-LPE

To study the pH responsive release profile of hemin and LOX of LHCa-LPE, LHCa-LPE (200 μL, LOX was labelled with FITC) was immersed in PBS (15 mL) at pH 6.5 and 7.4, followed by being incubated at 37 ^{o}C. At designed time intervals, the supernatants were collected to measure the amount of released hemin and LOX. The released hemin or LOX was determined by recording the characteristic absorbance of hemin at 384 nm or the FITC fluorescence (Ex. = 490 nm, Em. = 525 nm) by using a microplate reader (Bio-tek Synergy H1, USA), respectively.

Time-dependent catalytic capacity of LOX fueled by Lipiodol

To evaluate the catalytic capacity of LOX with Lipiodol as the substrate, lipid peroxidation probe of BODIPY-C11 was pre-solubilized in Lipiodol, followed by being mixed with $CaCO_3$, hemin, and LOX to obtain LHCa-LPE. Then, LHCa-LPE was incubated at room temperature under stirring. At determined time intervals, the emulsions (50 μL) were dissolved in ethanol, followed by being centrifugated and recording the fluorescence intensities of supernatants through a microreader (Ex. = 488 nm, Em. = 530 nm).

Cell experiments

Murine H22 hepatocellular carcinoma cell was obtained from Shanghai Zhongqiao Xinzhou Biological Technology Co., Ltd., and maintained in RPMI 1640 medium supplemented with 20% FBS and 1% penicillin/streptomycin. Rat N1S1 hepatocellular carcinoma cell was obtained from

Lishui Hospital of Zhejiang University as a gift, and maintained with DMEM high-glucose medium supplemented with 10% FBS and 1% penicillin/streptomycin. Human HepG2 hepatocellular carcinoma cells, murine NIH 3T3 embryo fibroblast cell lines, and human umbilical vein endothelial cell (HUVEC) were all purchased from the Cell Bank, Shanghai Institutes for Biological Sciences, Chinese Academy of Sciences. All the cell lines were cultured in the standard incubator under 5% CO_2 at 37 ^oC.

To determine a relatively safe concentration of Lipiodol, hemin, and LOX for the following cell experiments, N1S1 cells pre-seeded in 96-well plates at a density of 10^4 cells per well were incubated with a series of concentration of Lipiodol (100, 50, 25, 12.5, 6.25, 3.12, 1.56, 0.78, 0.39 μL Lipiodol in per mL of medium), hemin (200, 100, 50, 25, 12.5 μg mL^{-1}), and LOX (200, 100, 50, 25, 12.5 μg mL^{-1}) for 24 h before their viabilities determined by a commercial cell counting kit 8 (CCK-8) assay.

To evaluate the cell killing ability of the LOX fueled by Lipiodol in the presence or absence of hemin, N1S1 cells pre-seeded in 96-well plate (10^4 cells per well) were incubated with a series of concentrations of LOX, and fixed concentrations of Lipiodol (12.5 μL mL^{-1}) and hemin (12.5 μg mL^{-1}) for 24 h before their viabilities determined by CCK-8 assay.

To examine the catalytic capacity of LOX fueled by Lipiodol in inducing intracellular lipid peroxidation, N1S1 cells pre-seeded in 12-well plate (10^5 cells per well) were incubated with Lipiodol, LOX, hemin, or their combinations (Lipiodol = 12.5 μL mL^{-1}, hemin = 12.5 μg mL^{-1}, LOX = 50 μg mL^{-1}) for 6 h. Then, these cells were washed with PBS and incubated in the fresh medium containing BODIPY-C11 dye (1.5 μM) for 30 min before being imaged via confocal microscopy (CLSM, Zeiss, LSM 800). Besides, these N1S1 cells post various treatments and BODIPY-C11 staining were also analyzed by using a flow cytometer (BD, AccuriTM C6 Plus). Moreover, these N1S1 cells post different treatments as mentioned above were also incubated in the fresh medium containing DCFH-DA (20 μM) for 30 min before CLSM observation and flow cytometric analysis as aforementioned.

To confirm the ferroptotic cell killing pathway of such LOX fueled by Lipiodol and hemin, N1S1 cells pre-seeded in 12-well plate (10^5 cells per well) were incubated with LOX (50 μg mL^{-1}), hemin (12.5 μg mL^{-1}), and Lipiodol (12.5 μL mL^{-1}) in the presence or absence of Fer-1 (10 μM) and GSH (1 mM) for 6 h before being stained with BODIPY-C11 dye (1.5 μM) and analyzed via confocal microscopy and flow cytometry as aforementioned. Meanwhile, the cell

killing capacity of such catalytic mixtures towards N1S1 cells with same inhibitor treatments was also evaluated by determining their viabilities using CCK-8 assay.

To evaluate the pH-responsive cell killing effects of LHCa-LPE, 100 μL of LHCa-LPE was injected into the inserts of commercial transwell and were gently placed into a 24-well plate pre-seeded with N1S1 cells (5×10^4 cells) containing 1 mL of medium at pH 7.4 and 6.5. After a 24 h incubation, the cell viabilities were determined by a commercial CCK-8 assay.

Animal experiments

Female Balb/c mice (6-8 weeks), male Sprague-Dawley (SD) rats (300~350 g) were all purchased from Laboratory Animal Center of Soochow University, and used by following the protocols approved by Laboratory Animal Center of Soochow University. To establish the subcutaneous H22 tumor model, H22 cells (2×10^6) suspended in 50 μL of PBS were subcutaneously injected to the right flank of each Balb/c mouse. To establish the orthotopic N1S1 tumor model, N1S1 cells (6×10^6) suspended in 75 μL of PBS containing 30% Matrigel (Corning) were injected into the right lower liver lobe of each SD rat using a 25-gague syringe needle under anesthesia.

To assess the intratumoral retention behaviors of Lipiodol, Ca-LPE, H-LPE, and HCa-LPE, 12 mice bearing subcutaneous H22 tumors were randomly divided into four groups (n = 3) and then intratumorally injected with Cy5.5-labeled Lipiodol, Ca-LPE, H-LPE, and HCa-LPE at the same dose of Cy5.5, respectively. At the designed time intervals post injection (5 min, 2 h, 4 h, 8 h, 12 h, and 24 h), these treated mice were imaged under an IVIS® Lumina III in vivo fluorescence imaging system (PerkinElmer) to record the Cy5.5 fluorescence intensity. In addition, at 24 h, 48 h, and 72 h post injection, one mouse in each group was randomly picked out and sacrificed with their tumors collected and cryosectioned for CLSM observation.

To evaluate the intratumoral ROS levels of H22 tumors post different treatments, mice bearing subcutaneous H22 tumors (~110 mm^3) were randomly divided into five groups and then received the following intratumoral injections: group I, PBS; group II: Ca-LPE; group III: HCa-LPE; group IV: LCa-LPE; group V: LHCa-LPE. The intratumoral injection doses of LOX, hemin, Lipiodol, and $CaCO_3$ was 25 mg kg^{-1}, 10 mg kg^{-1}, 1.75 mL kg^{-1}, and 25 mg kg^{-1}, respectively. The volume of injected emulsions was 50 μL. Then, these treated mice were sacrificed with their tumors collected and cryosectioned for DCFH-DA staining at 24 h and 72 h

post intratumoral injection of various emulsions, followed by being subjected to CLSM observation.

To evaluate the therapeutic efficacy of LHCa-LPE, mice bearing subcutaneous H22 tumors (~110 mm^3) were randomly divided into six groups and received the following intratumoral injections: group I, PBS; group II: Ca-LPE; group III: HCa-LPE; group IV: LCa-LPE; group V: LHCa-LPE; group VI: Lipiodol-DOX. The intratumoral injection doses of LOX, hemin, Lipiodol, $CaCO_3$, and DOX was 25 mg kg^{-1}, 10 mg kg^{-1}, 1.75 mL kg^{-1}, 25 mg kg^{-1}, and 3 mg kg^{-1}, respectively. Since then, the length and width of each tumor and the body weight of each mouse were recorded every other day by using a Vernier caliper and digital balance, respectively. The tumor volumes were calculated by following the formula of tumor volume = (width*width*length)/2. When the tumor volume reached 1000 mm^3, these mice were presumed as dead. In addition, at two days post intratumoral injection, one mouse of each group was randomly picked out and sacrificed with their tumors collected for H&E staining.

To evaluate the TAFE treatment efficacy of LHCa-LPE toward orthotopic N1S1 HCCs, a total of 24 rats bearing orthotopic N1S1 hepatocellular carcinoma tumors (~360 mm^3) were randomly divided into four groups (n = 6) and treated as follows: group I, control; group II, transarterial embolization with Lipiodol; group III, transarterial embolization with LCa-LPE; group IV, transarterial embolization with LHCa-LPE. At day 0, the hepatic arteries of these rats were embolized with Lipiodol, LCa-LPE, and LHCa-LPE after being subjected to a 3.0-T MR imaging system. The embolized dosage of hemin, LOX, $CaCO_3$, and Lipiodol were 0.609 mg, 1.5 mg, 1.523 mg, and 150 μL, respectively. At 3, 7, 14 days post various treatments, these rats were intraperitoneally injected with commercial gadolinium contrast agent and then subjected to a 3.0-T MR imaging system for recording the tumor volumes. To further evaluate the therapeutic efficacies, one rat in each group was sacrificed for their tumors collected and cryosectioned for H&E and Ki67 staining at day 8.

Supplementary Figures

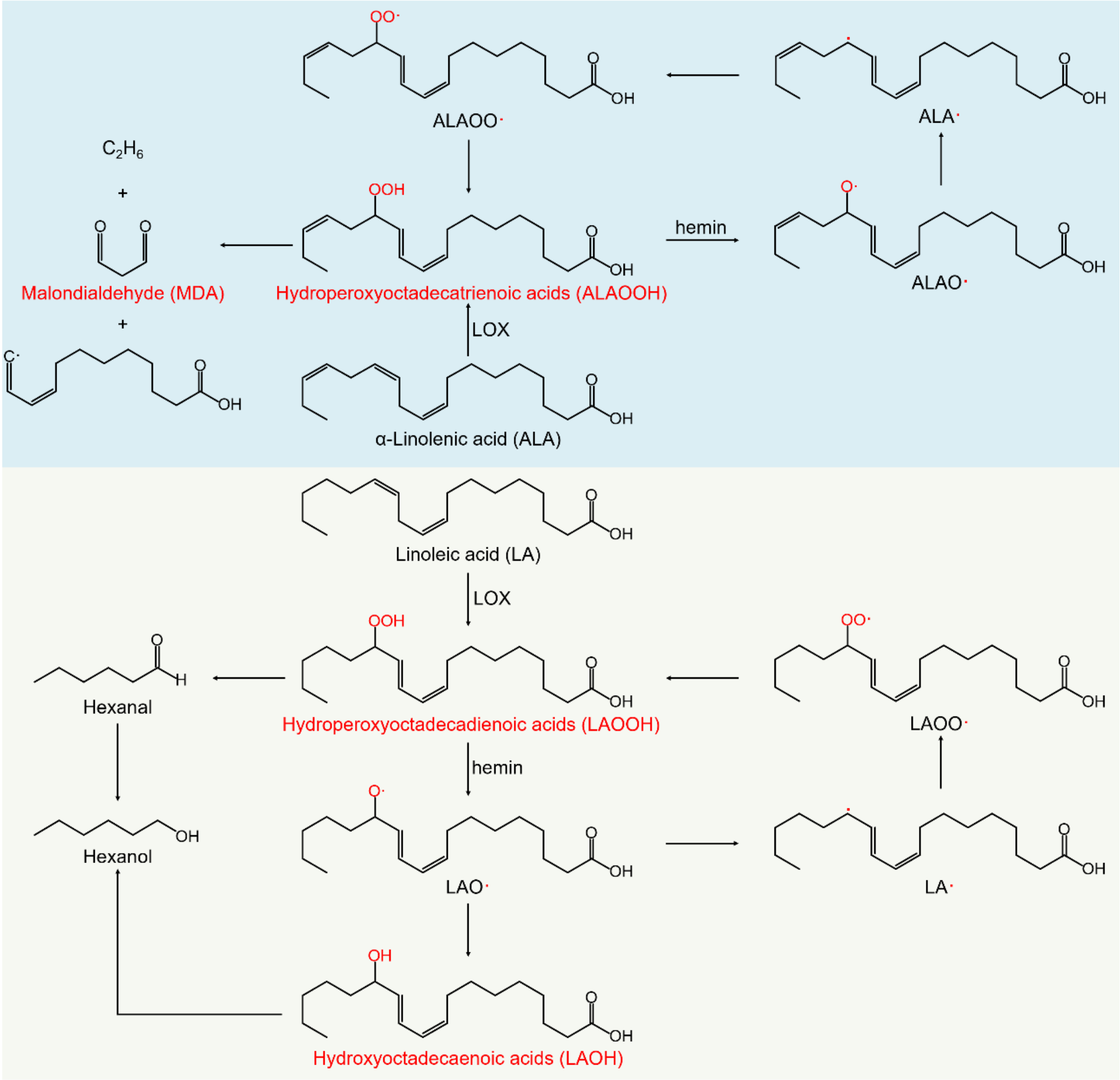

Scheme S1. Schematic illustration of the detailed reaction process of α-linolenic acid (ALA) or linoleic acid (LA) existing in Lipiodol catalyzed by LOX and hemin.

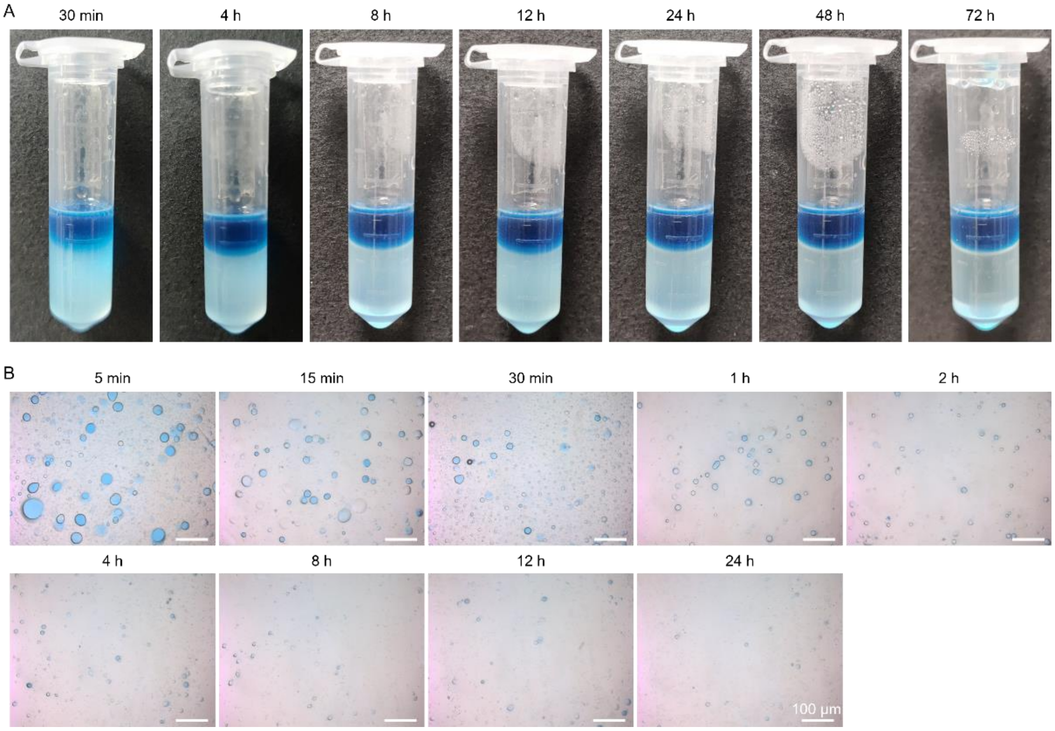

Figure S1. Time-dependent digital photographs (A) and optical microscopic images (B) of Lipiodol-water emulsions (methylene blue dissolved in aqueous-phase).

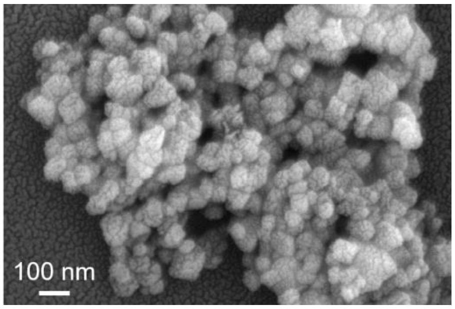

Figure S2. Representative SEM image of $CaCO_3$ nanoparticles.

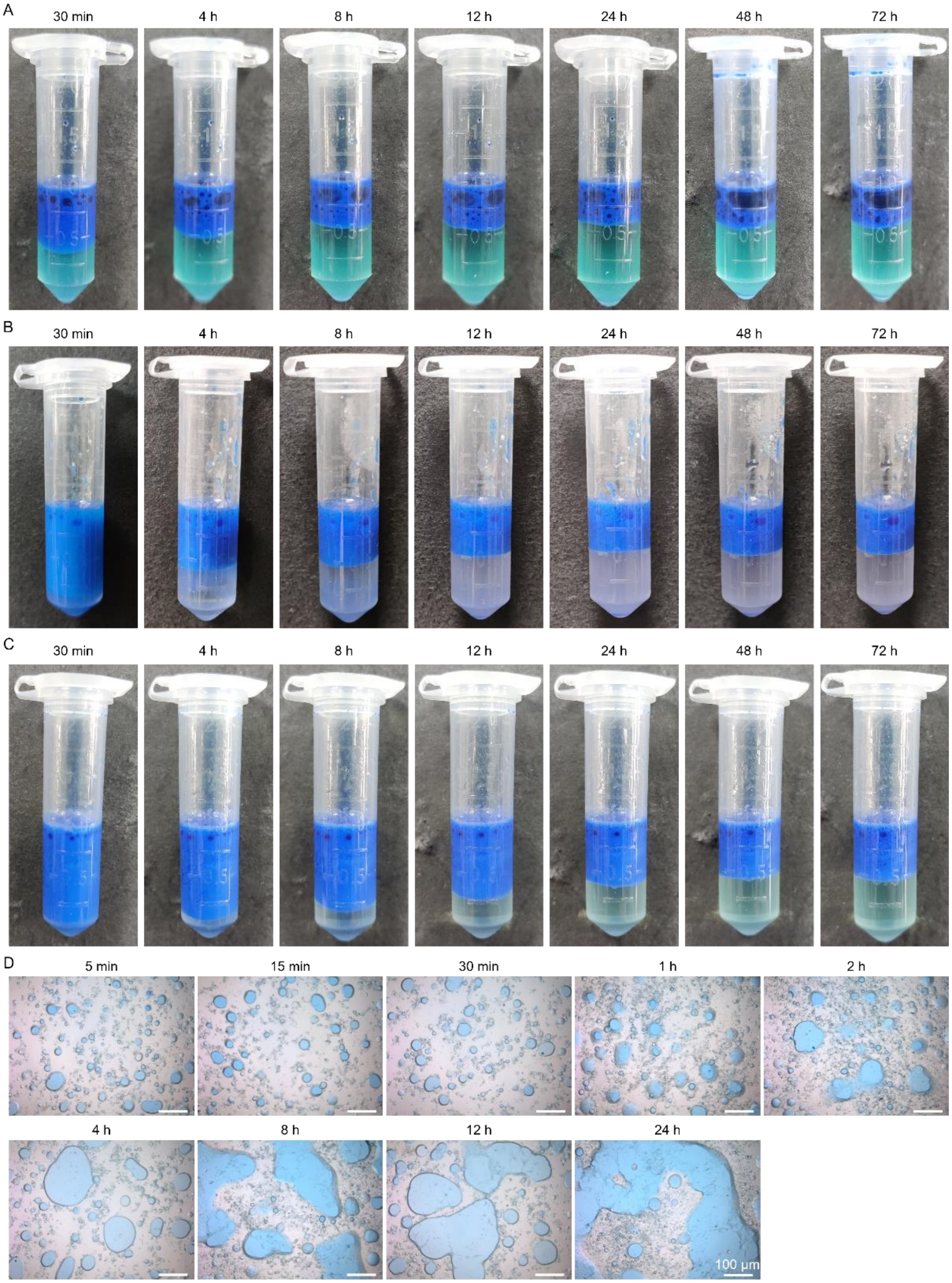

Figure S3. (A-C) Time-dependent digital photographs of Ca-LPE stabilized by $CaCO_3$ nanoparticles at a dose of 5 mg (A), 10 mg (B), and 20 mg (C), respectively. (D) Time-dependent optical microscopic images of Lipiodol emulsions stabilized by 10 mg of $CaCO_3$ nanoparticles (the blue of MB in aqueous droplets).

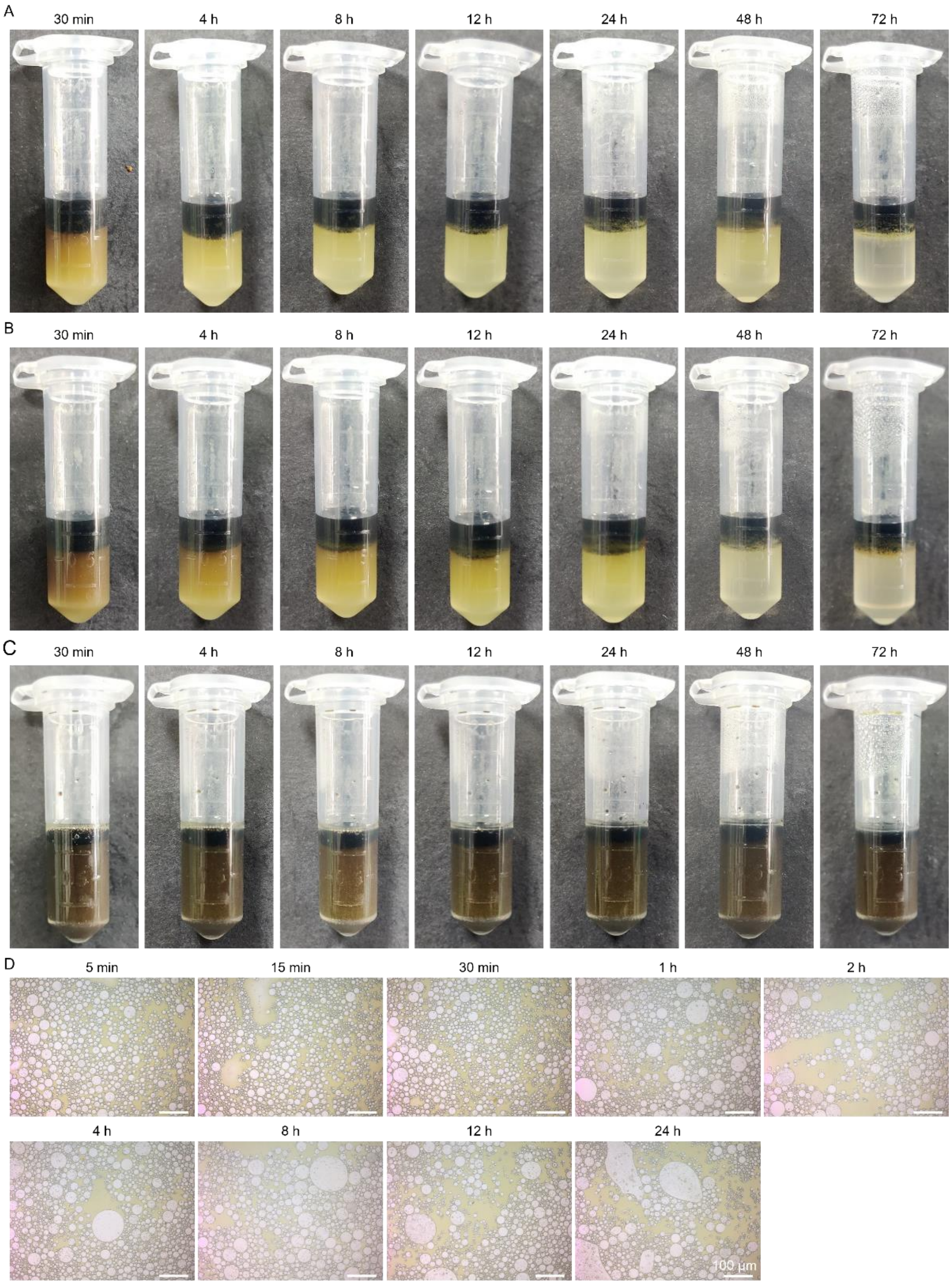

Figure S4. (A-C) Time-dependent digital photographs of H-LPE stabilized by hemin at 1 mg (A), 2 mg (B), and 4 mg (C), respectively. (D) Time-dependent optical microscopic images of Lipiodol emulsions stabilized by 4 mg of hemin.

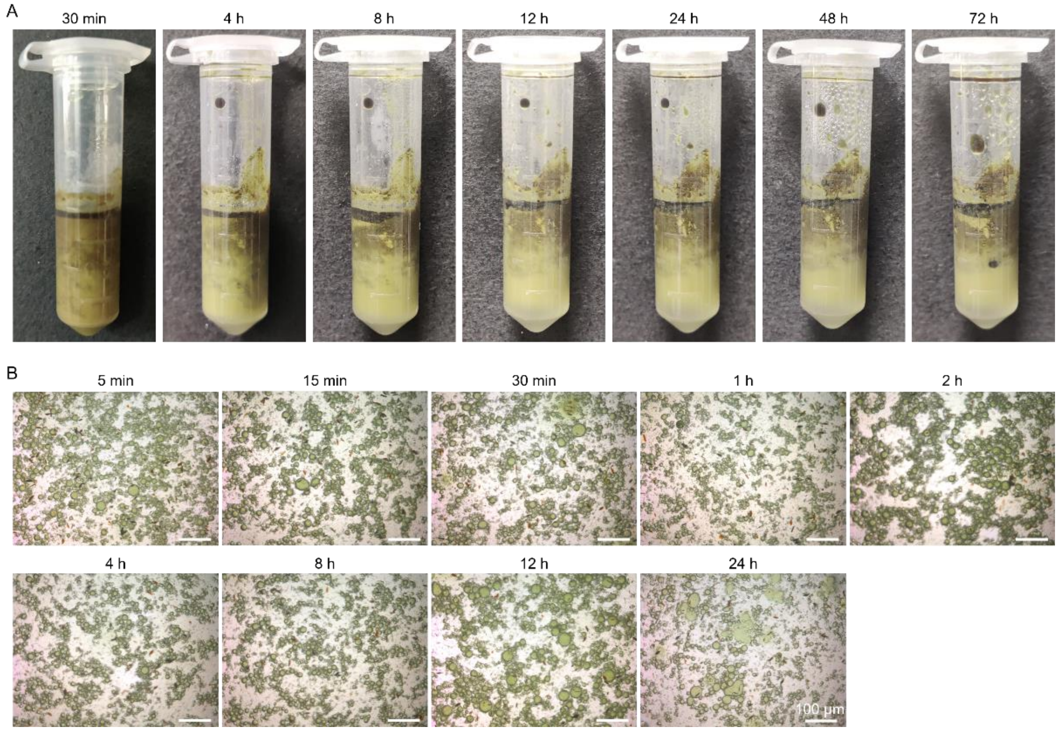

Figure S5. Time-dependent digital photographs (A) and optical microscopic images (B) of LHCa-LPE stabilized by 10 mg of $CaCO_3$ nanoparticles and 4 mg of hemin.

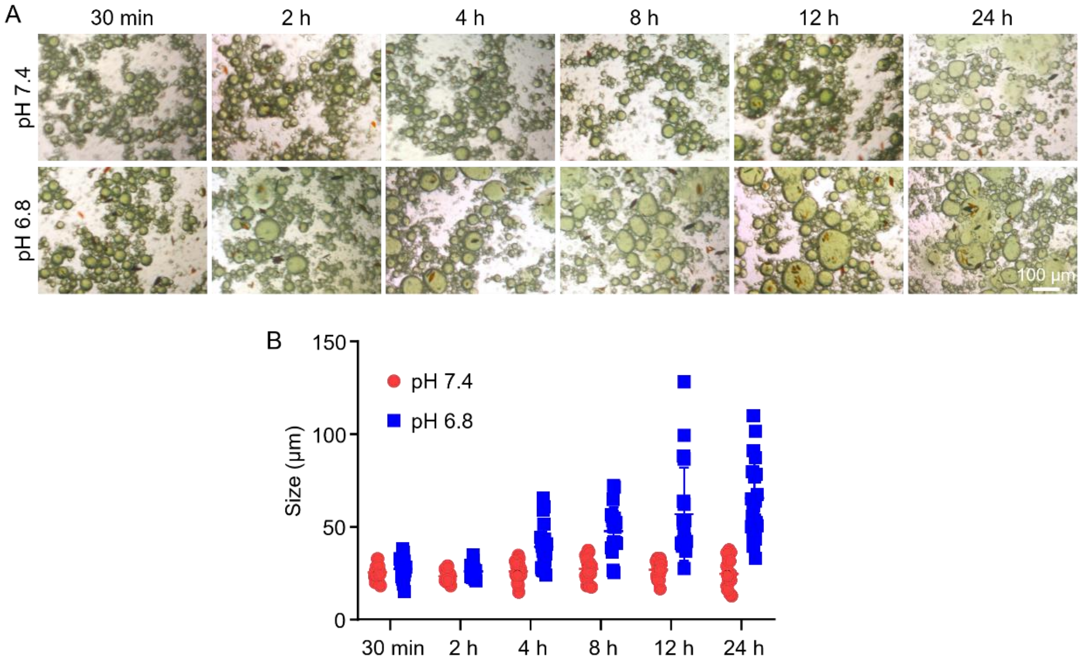

Figure S6. A) Time-dependent optical microscopic images of LHCa-LPE incubated at pH 7.4 or 6.5 buffers. B) The droplets size on the images shown in Figure S6A by using Nano Measurer 1.2 software.

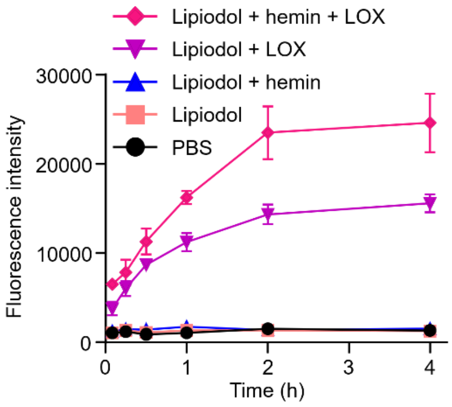

Figure S7. Time-dependent lipid peroxidation of Lipiodol via various treatments as indicated. Data are presented as the mean ± SD, n = 3 biologically independent samples.

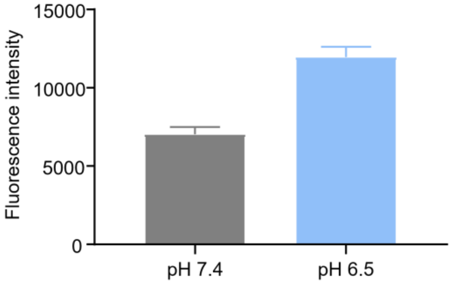

Figure S8. Lipid peroxidation of LHCa-LPE incubated in PBS at pH 7.4 or 6.5 for 4 h. Data are presented as the mean ± SD, n = 3 biologically independent samples.

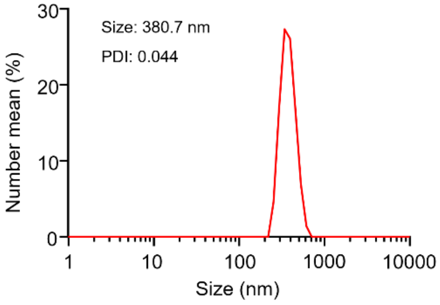

Figure S9. Hydrodynamic size distribution of Lipiodol micelles emulsified with Pluronic F-127.

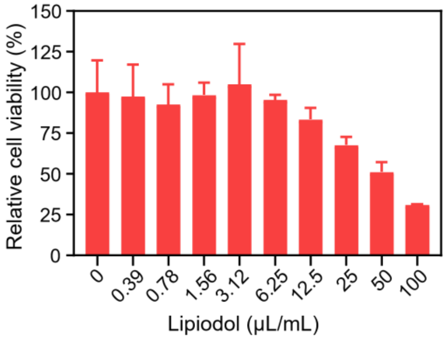

Figure S10. Relative cell viabilities of N1S1 cells incubated with different concentrations of Lipiodol for 24 h before being determined by CCK-8 assay. Data were represented as mean ± SD, n = 5 biologically independent samples.

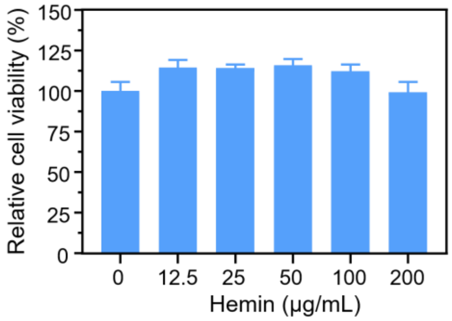

Figure S11. Relative cell viabilities of N1S1 cells incubated with a series concentration of hemin for 24 h before being determined by CCK-8 assay. Data were represented as mean ± SD, n = 5 biologically independent samples.

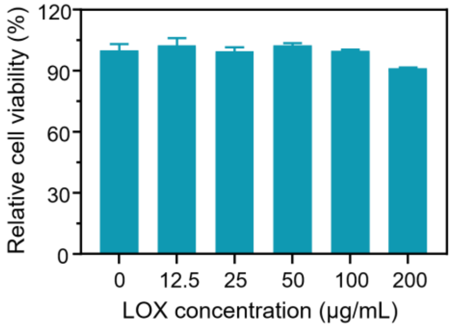

Figure S12. Relative cell viability of N1S1 cells incubated with a series concentration of LOX for 24 h before being determined by CCK-8 assay. Data were represented as mean ± SD, n = 5 biologically independent samples.

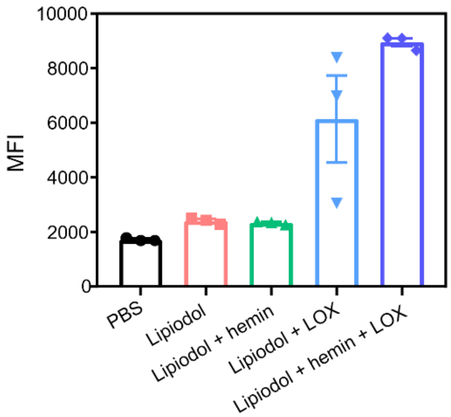

Figure S13. The mean fluorescence intensity of N1S1 cells post various treatments as indicated and stained with BODIPY-C11 via the flow cytometric analysis. Data were represented as mean ± SD, n = 3 biologically independent samples.

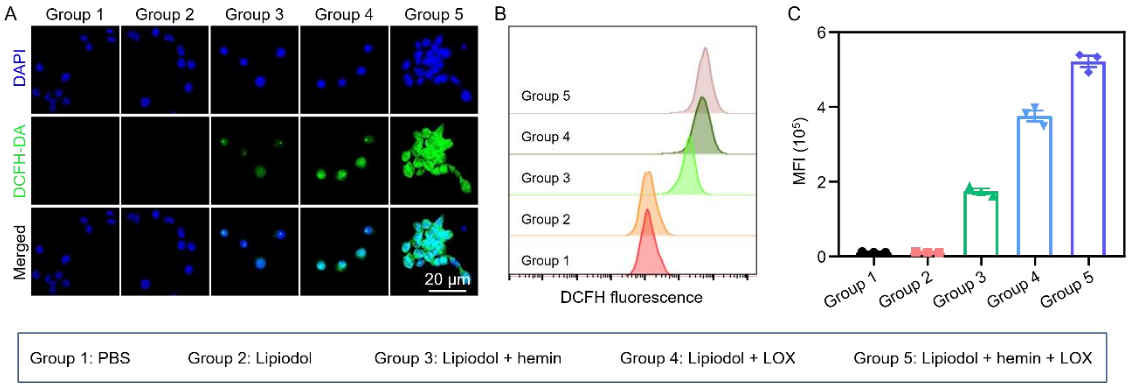

Figure S14. (A&B) Confocal images (A) and flow cytometric analysis (B) of intracellular ROS generation profiles of N1S1 cells incubated with various treatments as indicated for 6 h, respectively. (C) Semi-quantitative analysis of mean fluorescence intensity of these treated cells based on Figure B. Data were represented as mean ± SD, n = 3 biologically independent samples.

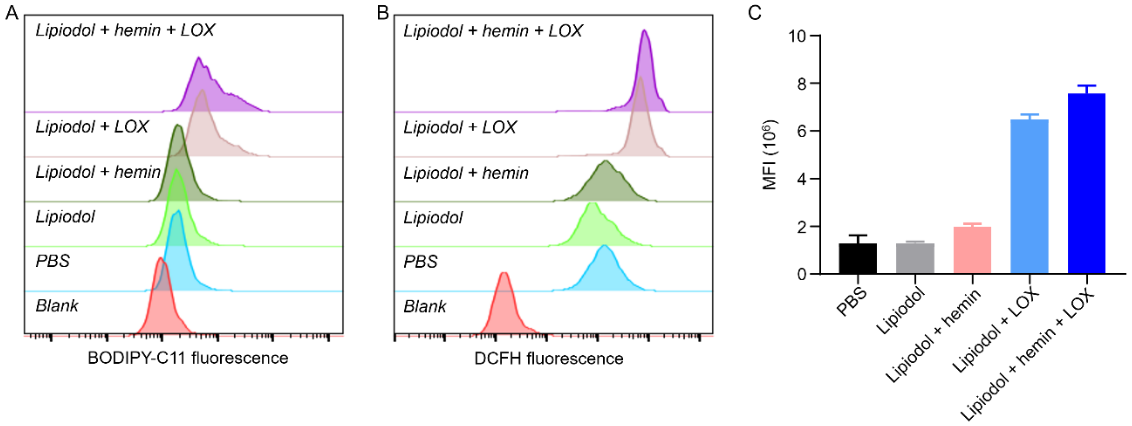

Figure S15. (A) Flow cytometric analysis of intracellular lipid peroxidation of HepG2 cells post various treatments as indicated, followed by being stained with BODIPY-C11 probe. (B&C) Flow cytometric analysis of intracellular ROS generation (B) and corresponding mean fluorescence intensity (C) of HepG2 cells post various treatments as indicated, followed by being stained with DCFH-DA probe. Data were represented as mean ± SD, n = 3 biologically independent samples.

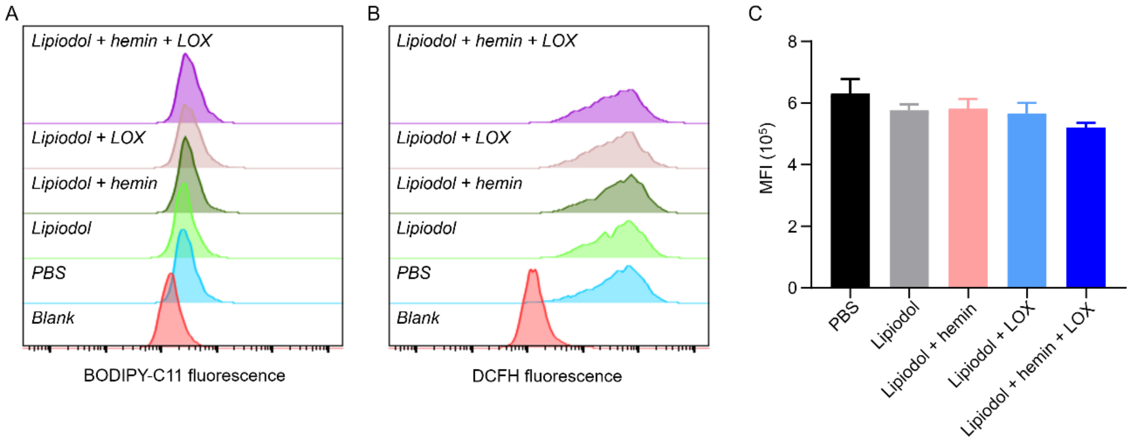

Figure S16. (A) Flow cytometric analysis of intracellular lipid peroxidation of NIH-3T3 cells post various treatments as indicated, followed by being stained with BODIPY-C11 probe. (B&C) Flow cytometric analysis of intracellular ROS generation (B) and corresponding mean fluorescence intensity (C) of 3T3 cells post various treatments as indicated, followed by being stained with DCFH-DA probe. Data were represented as mean ± SD, n = 3 biologically independent samples.

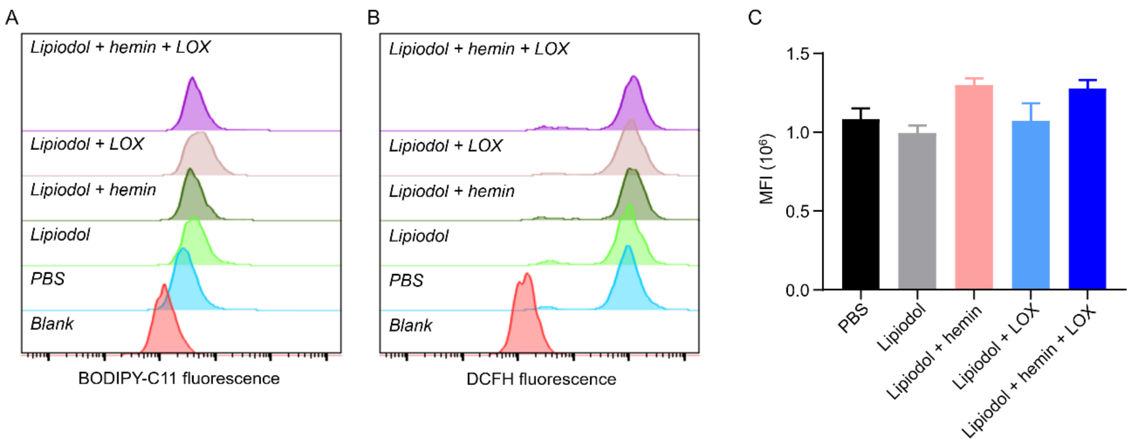

Figure S17. (A) Flow cytometric analysis of intracellular lipid of HUVEC cells post various treatments as indicated, followed by being stained with BODIPY-C11 probe. (B&C) Flow cytometric analysis of intracellular ROS generation (B) and corresponding mean fluorescence intensity (C) of HUVEC cells post various treatments as indicated, followed by being stained with DCFH-DA probe. Data were represented as mean ± SD, n = 3 biologically independent samples.

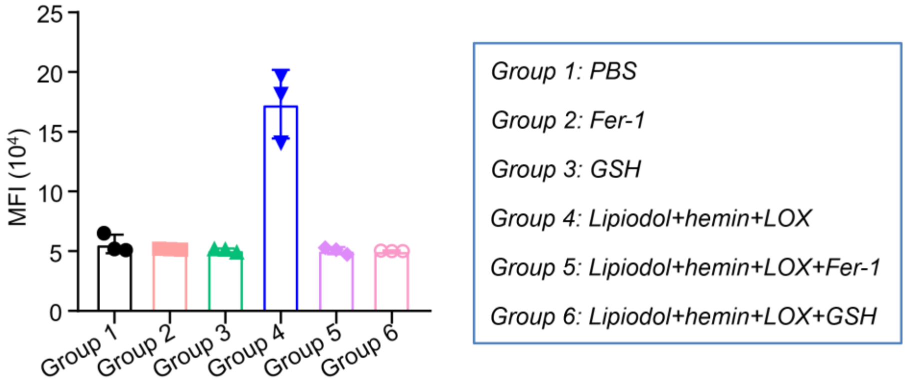

Figure S18. The mean fluorescence intensity of N1S1 cells post various treatments as indicated and stained with a lipid peroxidation probe of BODIPY-C11 via the flow cytometric analysis. Data were represented as mean ± SD, n = 3 biologically independent samples.

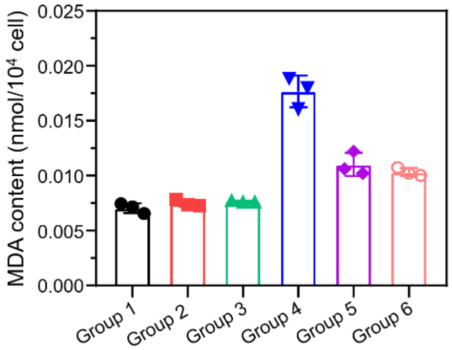

Figure S19. The malondialdehyde (MDA) content of N1S1 cells post various treatments (Group 1: PBS; Group 2: Fer-1; Group 3: GSH; Group 4: Lipiodol+hemin+LOX; Group 5: Lipiodol+hemin+LOX+Fer-1; Group 6: Lipiodol+hemin+LOX+GSH). Data were presented as mean ± SD, n = 3 biologically independent samples.

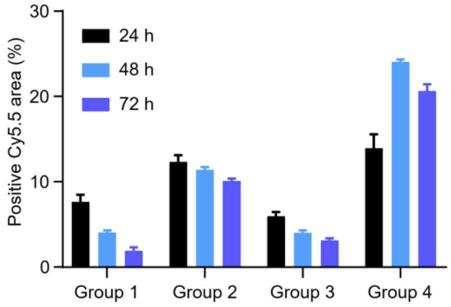

Figure S20. Semi-quantitative analysis of Cy5.5-positive areas based on the images shown in Figure 3D.

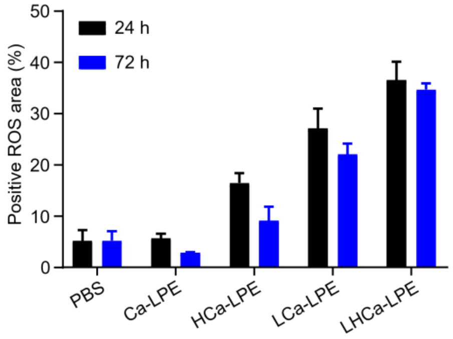

Figure S21. Semi-quantitative analysis of ROS-positive (DCFH fluorescence) areas based on the images shown in Figure 3E.

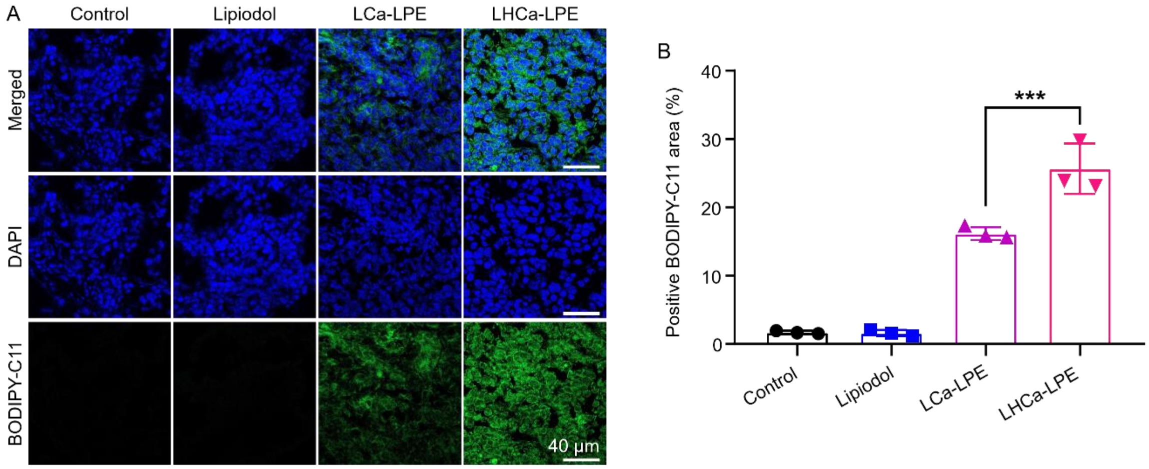

Figure S22. (A) Confocal images of tumor slices collected from N1S1 tumor-bearing rats at 3 days post various treatments as indicated. Those tumor slices were stained with BODIPY-C11 probe. (B) Semi-quantitative analysis of BODIPY-C11 positive areas based on the images shown in Figure S22A. *p* values were calculated by using ordinary one-way ANOVA. ***p<0.001.

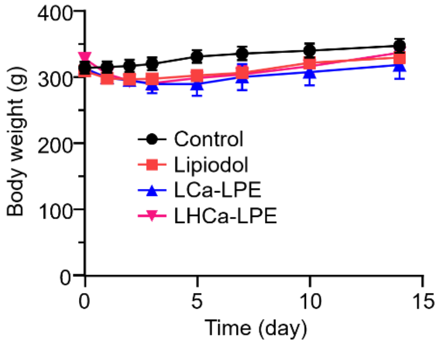

Figure S23. Average body weights of N1S1 tumor bearing-rats post various treatments as indicated. Data were represented as mean ± SD, n = 5 biologically independent samples.
